# The Effects of a Supermarket-Based Intervention on the Nutritional Quality of Private-Label Foods: A Prospective Study

**DOI:** 10.3390/nu12061692

**Published:** 2020-06-05

**Authors:** Daisy H. Coyle, Jason HY Wu, Gian Luca Di Tanna, Maria Shahid, Fraser Taylor, Bruce Neal, Helen Trevena

**Affiliations:** 1 The George Institute for Global Health, Faculty of Medicine, University of New South Wales, Sydney, NSW 2042, Australia; jwu1@georgeinstitute.org.au (J.H.W.); GDiTanna@georgeinstitute.org.au (G.L.D.T.); mshahid@georgeinstitute.org.au (M.S.); ftaylor@georgeinstitute.org.au (F.T.); bneal@georgeinstitute.org.au (B.N.); helen.trevena@sydney.edu.au (H.T.); 2 Menzies Centre for Health Policy, Sydney School of Public Health, Faculty of Medicine and Health, Charles Perkins Centre, University of Sydney, Sydney, NSW 2006, Australia

**Keywords:** supermarket, reformulation, private label, sodium, sugar, saturated fat, Health Star Rating

## Abstract

Private-label products, products owned by supermarkets, are a growing area of the food supply. The aim of this study was to assess the effect of an intervention that provided an Australian supermarket (‘intervention supermarket’) with comparative nutrition data to improve the healthiness of their private-label range. Between 2015 and 2016, the intervention supermarket received reports that ranked the nutritional quality of their products against competitors. Changes in the nutrient content (sodium, sugar, saturated fat, energy and Health Star Rating) of products from the intervention supermarket between 2015 and 2018 were compared against changes achieved for three comparators (private-label products from two other supermarkets and branded products). The intervention supermarket achieved a significantly greater reduction in the sodium content of their products relative to all three comparators, which ranged between −104 and −52 mg/100 g (all *p* < 0.05). Conversely, the three comparators each achieved a greater relative reduction in the sugar content of their products by between −3.5 and −1.6 g/100 g (all *p* < 0.05). One of the comparators also had a greater relative reduction in the saturated fat and energy content of their products compared to the intervention supermarket (both *p* < 0.05). There were negligible differences in the Health Star Rating of products between the intervention supermarket and comparators (all *p* > 0.05). Providing comparative nutrition information to a supermarket may be ineffective in improving the healthiness of their private-label products, likely due to competing factors that play a role in the decision-making process behind product reformulation and product discontinuation/innovation.

## 1. Introduction

Non-communicable diseases (NCDs), including diabetes and cardiovascular diseases, are the leading cause of death and disability globally [1]. In Australia, NCDs accounted for 89% of all deaths in 2016 [2] and are a major and growing economic burden on the health system [3,4]. Unhealthy diets are a leading modifiable risk factor for NCDs [5,6,7], driven by the availability of processed foods that frequently contain high quantities of added sodium, sugar and saturated fat [8,9]. Supermarkets dominate the retail food sector and, therefore, play an important role in the provision of processed foods in Australia and globally [10,11].

In Australia, supermarket sales accounted for 62% of household food expenditure between 2012 and 2013 [12]. The supermarket sector has been largely dominated by two chains, which have made up ~70% of total grocery sales for the past five years [13]. Supermarkets stock a large and diverse range of processed foods and beverages [6], which can be classified into two groups—(i) branded products and (ii) private-label products. Branded products are those owned by food companies and available for sale across multiple supermarket chains, whereas private-label products are owned by the supermarket and sold exclusively in their own stores, also known as ‘own-brand’ or ‘home-brand’ products [14].

Supermarkets have invested in their private-label range to meet consumer demands for price, value and quality [15]. Today, many supermarkets have segmented their private-label products to provide value, mid-range and premium options [14], and have increased their product range within categories. Consequently, private-label products have experienced increased sales and shifts in consumer perceptions of their products, from ‘low cost, low quality’ to adequate quality [14,15,16]. Australian private-label foods and beverages are predicted to make up 26% of the total share of grocery sales by 2021 [17]—almost double the volume in 2009 [18]. As such, interventions that improve the healthiness of private-label foods and beverages could support consumers to make healthier choices [19,20] with potentially large impacts on population health [21].

This study reports on a co-developed intervention between public health nutrition researchers at The George Institute for Global Health and an Australian supermarket (hereafter, ‘intervention supermarket’). The aim of the intervention was to provide the intervention supermarket with comparative nutrition data to improve the healthiness of their private-label range over a period of 24 months. The objective of this paper was to assess the effect of this co-developed intervention on the healthiness of the supermarkets private-label products as measured by changes in sodium, sugar, saturated fat and energy and Health Star Rating (a voluntary, government-led front-of-pack nutrient profiling system [22]). To gain insight into the changes achieved in the context of the greater retail environment, we compared nutrient differences over time for the intervention supermarket relative to changes achieved for three comparator groups.

## 2. Materials and Methods

### 2.1. Intervention

The intervention was co-designed by researchers at The George Institute and the nutrition team at the intervention supermarket to help identify opportunities to improve the nutritional quality of their private-label products over a period of 24 months. Expectations relating to the process of the intervention were established and documented in a project set-up stage and aligned with principles for public–private partnerships [23,24]. This included agreement on roles and responsibilities, pre-defined timelines, project governance, issue management and agreement on product categories included within scope of the intervention. During the course of the intervention, fortnightly meetings of approximately one hour were held between The George Institute and the supermarket nutrition team. Working to a pre-agreed agenda, action items were discussed, documented and shared, and any issues were raised and resolved. In addition to this, there was communication via email and telephone in between meetings and formal presentations at the start and end of the intervention. 

A total of 150 product categories were selected on the basis of (i) priority areas for the intervention supermarket, (ii) the stage in the product life-cycle and iii) alignment with public health initiatives [25]. A detailed list of product categories included in the intervention are provided in Table S1. Products were grouped into major product categories (e.g., bread and bakery products), minor categories (e.g., biscuits), and subcategories (e.g., savoury biscuits) using the FoodSwitch categorisation system, which is based on original work of the Global Food Monitoring Group [26]. All value, mid-range and premium options were included.

The intervention was delivered over a nine-month period, from April 2015 to January 2016. Thirty-five comparative benchmark nutrition reports covering 12 major, 30 minor and 150 detailed subcategories of foods and beverages were generated using a co-designed report template. The development of the reports was governed by a project manager and the reports and analysis were provided by public health researchers and Accredited Practising Dietitians at The George Institute. The reports were delivered to the nutrition team lead at the intervention supermarket and due dates for the reports were scheduled in the contract and were sent in batches of around four reports per month.

Each report detailed the number of products per manufacturer and provided a summary of nutritional composition [sodium (mg/100 g), sugar (g/100 g), saturated fat (g/100 g) and energy (kJ/100 g)] and Health Star Rating (stars) including the observed mean, median, and maximum and minimum level for each nutrient for all products available for sale across each manufacturer. The reports also ranked and compared the private-label products of the intervention supermarket against all competitor products according the nutrient content and Health Star Rating. The reports did not provide advice or recommendations for how the intervention supermarket should use or interpret the information provided.

### 2.2. Data Source for the Intervention—The Product Category Reports

The 2015 FoodSwitch monitoring database [27] was used to generate the product category reports. The entire database holds data for ~100,000 processed foods and beverages from a cross-sectional survey of packaged and barcoded foods that are available for sale each year, from August to October, from four major supermarkets (ALDI, Coles, IGA and Woolworths) in Sydney, Australia [27]. The nutrient information is obtained directly from the mandatory nutritional information panel and includes information regarding the nutrient content per 100 g/mL and per serve for all macro-and micro-nutrients listed. Other variables include the product name, manufacturer name, package size, nutrient claims and the Health Star Rating. The Health Star Rating rates the overall healthiness of a food according to the nutrient profile on a scale of 0.5 (least healthy) to five stars, in increments of 0.5 stars. The number of stars is based on a nutrient profiling algorithm, which has been described elsewhere [22]). The Health Star Rating was taken from the front of pack if displayed and if not, it was calculated in the FoodSwitch system using the Health Star Rating algorithm (using proxy values derived from the database where necessary) [27]. As described previously, data are entered according to rigorous and standardised procedures including screening for outliers and checks for data entry accuracy [27].

### 2.3. Comparators

Two Australian supermarkets (Comparators A and B) also selling private-label products were assigned as comparators. These supermarkets were selected as comparators as they had the largest number of private-label products in the FoodSwitch database and were largely comparable to the intervention in terms of number of products available for sale. All branded products available in the FoodSwitch database were also included in this study as a comparator (Comparator C).

### 2.4. Outcomes

As this was a trial, we a priori defined the primary outcome as the mean overall change in the sodium (mg/100 g), sugar (g/100 g), saturated fat (g/100 g) and energy (kJ/100 g) content, and Health Star Rating (stars) between 2015 and 2018.

### 2.5. Data Source—Intervention Outcomes

Nutrient data were sourced from the FoodSwitch monitoring databases for the years 2015 (*n* = 20,689) and 2018 (*n* = 22,165) [26]. Products were excluded if: (i) they were not targeted as part of the intervention (2015 = 12,378; 2018 = 12,895), (ii) they were missing energy, sodium, sugar and/or saturated fat information (2015 = 1037; 2018 = 241) or (iii) they were duplicate products (i.e., same products available in different pack sizes) (2015 = 571; 2018 = 601).

To account for repeated measures for products available in both years, all products were classified as either ‘matched’ or ‘unmatched’. Matched products were those available in both 2015 and 2018 based on barcode and product name. Unmatched products were those available in either 2015 or 2018 only. This disaggregation also allowed us to assess the extent of nutrient changes driven by product reformulation (matched products) versus product discontinuation and product innovation (unmatched products).

### 2.6. Statistical Analysis

The number and proportion of products available in each year and the percentage change in the number of products available over time were calculated.

The primary analysis involved comparing the mean change in nutrient content (sodium, sugar, saturated fat and energy) and Health Star Rating over time (2015 versus 2018) between the intervention supermarket and each of the three comparators. Dependent *t*-tests were used to assess mean differences in nutrient content over time (2015 versus 2018) for the matched products and independent *t*-tests were used to compare changes for unmatched products. Fixed effect meta-analyses were used to assess the change in nutrient content and Health Star Rating over time by pooling results for the matched and unmatched products. This was weighted to reflect the proportions of products available in 2018. Differences between the intervention and each of three comparators were calculated using summary estimates and standard errors from the meta-analyses using 100,000 Monte Carlo simulations to estimate uncertainty intervals for each nutrient and Health Star Rating.

To explore the potential impact of the nutrient content at baseline, we compared the mean nutrient content (sodium, sugar, saturated fat and energy) and Health Star Rating between the intervention supermarket and each of the three comparators for products available in 2015. Differences across groups were estimated using 1-factor ANOVA tests with Tukey’s honest significance difference test post-hoc analyses.

We also explored the nutrient content post-intervention by comparing the mean nutrient content (sodium, sugar, saturated fat and energy) and Health Star Rating between the intervention supermarket and each of the three comparators for products available in 2018. Differences between the intervention supermarket and each of the three comparators were estimated using 1-factor ANOVA tests with Tukey’s honest significance difference test post-hoc analyses.

To investigate the potential mechanisms in which the intervention supermarket achieved changes to the mean nutrient content (sodium, sugar, saturated fat and energy) and Health Star Rating between 2015 and 2018, we explored nutrient changes across both matched and unmatched products. Independent *t*-tests were used to compare changes over time for unmatched products and dependent *t*-tests were used for matched products. We also investigated overall change using a summary estimate from a fixed effect meta-analysis that pooled matched and unmatched products (and was weighted to reflect the proportions of products available in 2018). As supplementary analyses, descriptive statistics were used to describe the absolute change and percentage change in nutrient content over time within each group, by product category. Since sample sizes were sufficiently large to not require assumptions of normality, analyses and reporting are based upon mean values and parametric tests [28]. Two-sided *p*-values of <0.05 were considered statistically significant. To account for multiple testing, we applied the Benjamini–Hochberg procedure to decrease the false discovery rate. All statistical analyses were performed using Stata 15.0 (Stata Corp, College Station, TX, USA).

## 3. Results

In total, there were 6703 products analysed at baseline in 2015 and 8428 products in 2018. Across the four groups, Comparator C had the largest number of products in both years (2015 = 5026; 2018 = 6042), followed by Comparator B (2015 = 661; 2018 = 922), Comparator A (2015 = 596; 2018 = 899) and the intervention supermarket (2015 = 420; 2018 = 565).

The total number of products in each category for all years ranged from 35 for special foods to 1663 for bread and bakery products (See Table 1). With the exception of non-alcoholic beverages which decreased by 8.2%, the proportion of products in the other 11 categories all increased over time by between 2.9% and 69.6%.

### 3.1. Mean Nutrient Content in 2015 between the Intervention Supermarket and Comparators

**Figure 1:**
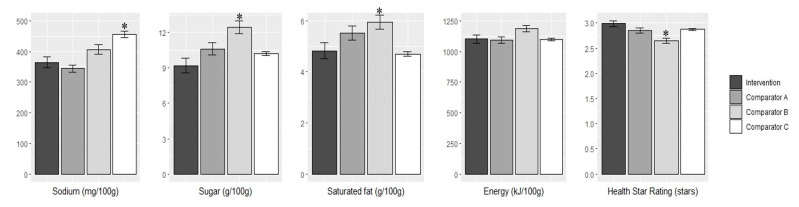
Comparison of the mean nutrient content [sodium (mg/100 g), sugar (g/100 g), saturated fat (g/100 g) and energy (kJ/100 g)] and Health Star Rating in 2015 between the intervention supermarket and comparators. Errors bars indicate the standard error (SE). Differences across groups were estimated using 1-factor ANOVA tests with Tukey’s honest significance difference test post-hoc analyses. * indicates significance difference compared to intervention supermarket (*p* < 0.05).

In 2015, products from the intervention supermarket had a substantially lower sodium content compared with products from comparator C (mean difference: −91 mg/100 g; 95% CI: −8, −174; *p* = 0.03, Figure 1). However, no differences were observed between the intervention and comparators A and B. Products from the intervention supermarket had a lower sugar content in 2015 compared with comparator B (mean difference: −3.3 g/100 g; 95% CI: −1.2, −5.3; *p* < 0.001) but there were no differences between the intervention and comparators A and C. Similarly, products from the intervention supermarket had a lower saturated fat content compared with comparator B (mean difference: −1.1 g/100 g; 95% CI: −0.1, −2.2; *p* = 0.01) with no differences between the intervention and comparators A and C. In 2015, products from the intervention supermarket also had a higher Health Star Rating compared with products from comparator B (mean difference: +0.3 stars; 95% CI: +0.1, +0.5; *p* = 0.01) with no differences between the intervention and comparators A and C. In 2015, there were no differences in the mean energy content between the intervention supermarket and any of the comparators (Figure 1).

### 3.2. Differences in the Mean Change in Nutrient Content between 2015 and 2018 between the Intervention Supermarket and Comparators

**Figure 2:**
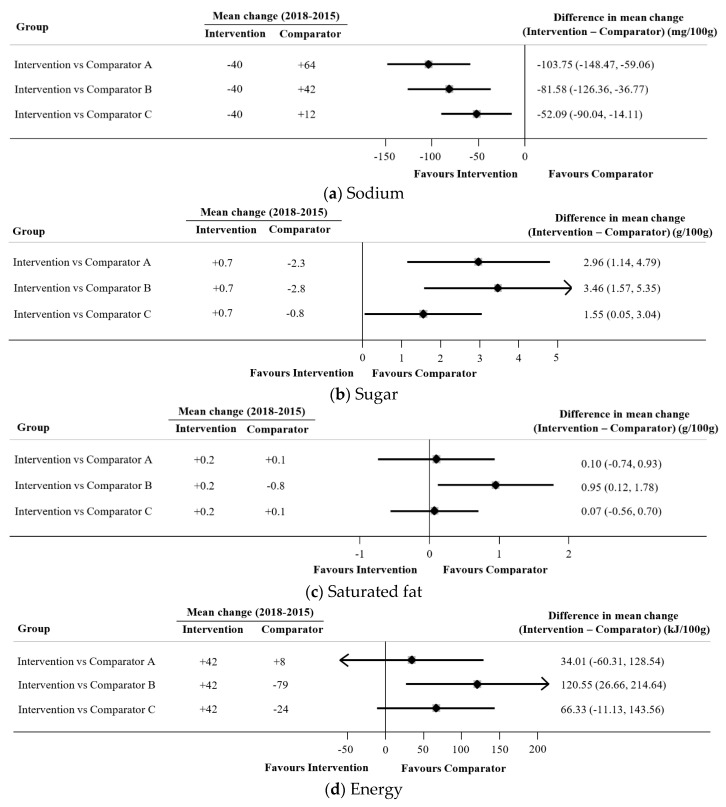

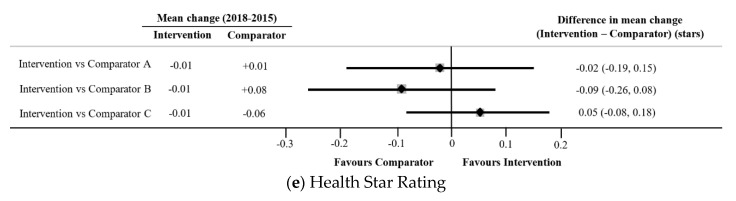
Differences in mean change in nutrient content (sodium (mg/100 g), sugar (g/100 g), saturated fat (g/100 g) and energy (kJ/100 g)) and Health Star Rating (stars) between 2015 and 2018 across the intervention supermarket and comparators Fixed effect meta-analyses were used to assess the change in nutrient content over time within each group. Each meta-analysis was weighted to reflect the proportions of products available in 2018. Differences across groups were then calculated using summary estimates and standard errors from the meta-analyses using 100,000 Monte Carlo simulations for each nutrient content.

The intervention supermarket had a greater reduction in the mean sodium content of its products over time relative to each of the three comparators (all *p* < 0.05) by between −104 and −52 mg/100 g (Figure 2a). Conversely, compared to the intervention supermarket, the three comparators each achieved a greater relative reduction in the mean sugar content of their products by between −3.5 and −1.6 g/100 g (all *p* < 0.05) (Figure 2b). Compared to the intervention supermarket, comparator B had a greater reduction in the saturated fat content of its products over time (−1.0 g/100 g; 95%CI: 0.1, 1.8) (*p* < 0.05) (Figure 2c). Similarly, comparator B had a greater relative reduction (*p* < 0.05) in the energy content of its products over time compared with the intervention supermarket (−121 kJ/100 g; 95% CI: −27, −215) (Figure 2d). There were no differences observed in the mean change in Health Star Rating over time between the intervention and any comparators (all *p* > 0.05) (Figure 2e).

### 3.3. Mean Change in Nutrient Content between 2015 and 2018 for Products in the Intervention Supermarket

**Figure 3:**
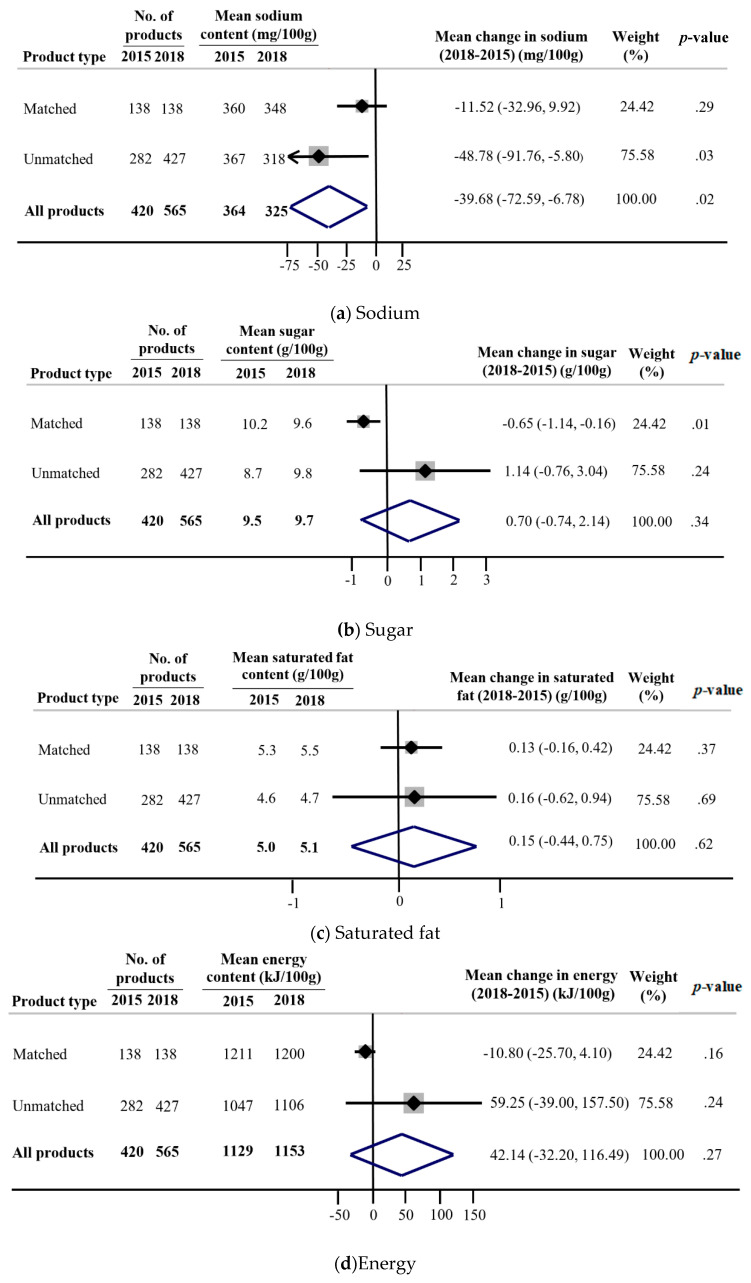

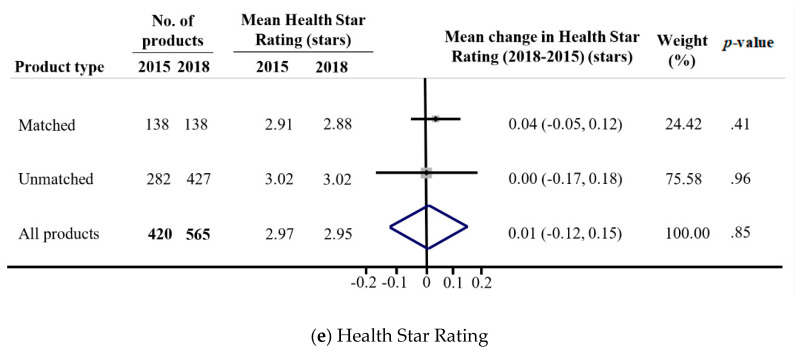
Mean change in nutrient content [sodium (mg/100 g), sugar (g/100 g), saturated fat (g/100 g) and energy (kJ/100 g)] and Health Star Rating between 2015 and 2018 for products in the intervention supermarket. Matched products are products available in both 2015 and 2018 and unmatched products are those available in either 2015 or 2018. Dependent *t*-tests were used to assess mean differences in nutrient content over time (2015 versus 2018) for the matched products and independent *t*-tests were used to compare changes over time for unmatched products. Differences in the nutrient content over time were estimated separately for matched and unmatched products and the overall changes were obtained using a summary estimate from a fixed effect meta-analysis. Each meta-analysis was weighted to reflect the proportions of products available in 2018.

A total of 985 products were available from the intervention supermarket between 2015 and 2018. Of these, 276 were matched products (138 in each year) and 709 were unmatched products, available only in 2015 (*n* = 282) or 2018 (*n* = 427). The overall mean sodium content was 10.7% lower in 2018 compared to 2015 (mean difference: −40 mg/100 g; 95% CI: −73, −7 mg/100 g; *p* < 0.02). For unmatched products, there was a 13.4% reduction in the mean sodium content (−49 mg/100 g; 95% CI: −92, −6; *p* = 0.03). No changes over time were observed for matched products (Figure 3a). Across specific product categories, over half (7 out of 12) had a numerical reduction in the mean sodium content (Table S2). The largest absolute reductions were found for meat and meat products (−195 mg/100 g), edible oils and oil emulsions (−133 mg/100 g) and cereal and grain products (−114 mg/100 g). The overall mean sugar content did not differ between 2015 and 2018 (*p* = 0.62). However, there was evidence to suggest that reformulation of products led to a sugar reduction, as the mean sugar content decreased significantly for matched products by 5.9% (−0.7 g/100 g; 95% CI: −1.1, −0.2; *p* = 0.01). No changes over time were observed for unmatched products (Figure 3b). Numerical reductions in mean sugar were observed for five of the twelve product categories, with the greatest reductions observed for sauces, dressings, spreads and dips (−1.4 g/100 g), convenience foods (−0.8 g/100 g) and bread and bakery products (−0.7 g/100 g) (Table S3). The overall mean saturated fat content, energy content and Health Star Rating did not change between 2015 and 2018, and no differences were observed for either matched or unmatched products (Figure 3c–e) (Tables S4–S6).

### 3.4. Comparison of the Mean Nutrient Content in 2018 between the Intervention Supermarket and Comparators

**Figure 4:**
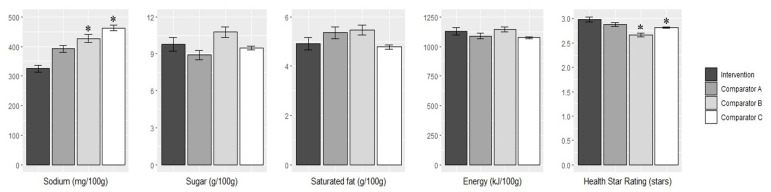
Comparison of the mean nutrient content (sodium (mg/100 g), sugar (g/100 g), saturated fat (g/100 g) and energy (kJ/100 g)) and Health Star Rating (stars) in 2018 between the intervention supermarket and comparators. Errors bars indicate the standard error (SE). Differences across groups were estimated using 1-factor ANOVA tests with Tukey’s honest significance difference test post-hoc analyses. * indicates significance difference compared to intervention supermarket (*p* < 0.05).

In 2018, the mean sodium content of products from the intervention supermarket was 325 ± 268 mg/100 g. This was significantly lower than products from comparator B (mean difference: −102 mg/100 g; 95% CI: −16, −188; *p* = 0.01) and comparator C (−137 mg/100 g; 95% CI: −66, 208; *p* < 0.001) but not comparator A (−67 mg/100 g; 95% CI: −153, +19; *p* = 0.2) (Figure 4).

Similarly, the mean Health Star Rating of products from the intervention supermarket in 2018 was 2.98 ± 1.18 stars. This was higher than products from comparator B (mean difference: +0.33 stars; 95% CI: +0.16, +0.49; *p* = 0.01) and comparator C (+0.17 stars; 95% CI: +0.03, +0.31; *p* < 0.001) but not comparator A (+0.1 stars; 95% CI: −0.1, +0.3; *p* = 0.4).

There were no differences between the intervention and any of the comparator groups in the mean sugar, saturated fat or energy content of products in 2018 (Figure 4).

### 3.5. Adjustment for Multiple Testing

After adjustment—by the Benjamini–Hochberg procedure—of all the *p*-values calculated in this study and keeping a false discovery rate at 10%, we have found no differences in the results in terms of statistical significance.

## 4. Discussion

Between 2015 and 2018, we identified a significant reduction in the mean sodium content of products in the intervention supermarket. However, no changes were observed for the sugar, saturated fat and energy content, and Health Star Rating. When changes to the nutrient content and Health Star Rating over time were compared against changes made by each of the three comparators, the intervention supermarket had a greater reduction in sodium over time. However, the results for sugar, saturated fat and energy were mostly in favour of the comparators. In 2018, the intervention supermarket had a lower sodium content and a higher Health Star Rating relative to two of the three comparators, with no differences across groups for sugar, saturated fat and energy.

At baseline, products from the intervention supermarket had less potential for improvement compared with comparators given their products generally had a lower sugar and saturated fat content and a higher Health Star Rating. As such, the greater relative reductions in sugar and saturated fat achieved by comparators was largely a consequence of these groups moving these nutrients down towards the levels already achieved by the intervention supermarket. Moreover, the comparative nature of the reports and the fact that the intervention supermarket generally had healthier products at baseline may have only emphasised to the nutrition team that their products were already of a greater nutritional quality thus reducing motivation to create improvements. Potentially different findings would have been observed if the intervention had focused on providing specific goals to improve the absolute quality of their private-label products, rather than in comparison with competitors.

The lack of consistent improvements achieved by the intervention supermarket in terms of the nutritional quality of their products may have also been influenced by a number of other factors. Over recent years, there has been intense competition between supermarket retailers in Australia as they continue to expand their private-label range [13]. This is likely to have generated competing priorities for supermarkets, influencing their decision-making process behind product reformulation, product discontinuation and new product development. This may have included considerations for cost, convenience and product sales as well as the taste and flavour of a product [29,30,31]. As a consequence, this may have reduced their emphasis on the nutritional quality. Moreover, it appears that the intervention supermarket prioritised sodium reduction, which may have been driven by public health initiatives around the time of the intervention. The previous federal government initiative, the Food and Health Dialogue [25,32], which ran from 2009 to 2015, had a specific focus on sodium including voluntary reformulation targets for a range of food categories. Similarly, its successor, the Healthy Food Partnership [33,34], established in late 2015, also has a focus on sodium reduction. However, the Healthy Food Partnership voluntary sodium reformulation targets were only released in 2020. Despite no actionable government policies at the time of the intervention, the long-term focus of sodium as part of government and non-government initiatives [35] may have motivated the intervention supermarket to prioritize this nutrient over others [36].

Previous literature has demonstrated that food companies employ different strategies to change the nutritional composition of their products depending on the nutrient and food category [37]. Our analysis of the nutritional changes achieved by the intervention supermarket according to matched and unmatched products supports this research. The intervention supermarket achieved greater reductions to the sodium content of its products due to formulation of new products and discontinuation of old product lines, rather than due to product reformulation. On the contrary, reductions to the sugar content were largely achieved by reformulation. The difference for matched and unmatched products for these nutrients suggests that both are valid strategies for improving the food supply. As such, it reinforces the importance of promoting both approaches to encourage food companies to improve the nutritional quality of their packaged foods and beverages [38]. As the market share of private-label products continues to grow [18,20] and as negative consumer preconceptions of inferior quality continue to be overturned [36], supermarkets will have increasing potential to play a significant role in improving healthiness of the food supply [39]. It is important that research continues to monitor the nutritional composition of the packaged food supply to track changes to nutrient levels, particularly with the high volume of new private-label products entering the market [40].

Public–private partnerships have been recommended globally, including as part of the United Nation Sustainable Development Goals [41], in order to help address public health challenges [24,42]. Whilst a number of public–private partnerships between large food companies and non-governmental organisations have been undertaken for many years to help tackle global issues [24], there are few examples within the supermarket sector, particularly in the Australian context [43]. This is an important area of research given the potential for supermarkets to have a positive impact on the Australian food supply given their considerable and ever-growing market share via their private-label products [44,45]. The limited overall improvement achieved by the intervention supports previous research suggesting that public–private partnerships with the food industry are largely ineffective without government regulation (or threat of regulation) [46,47].

Our study has several strengths. Firstly, the intervention was underpinned by evidence-based principles of public–private partnerships. This includes clearly defined goals that provide benefit to the public, project governance, clear objectives, defined baseline data to monitor progress and ongoing and transparent communication [23]. Secondly, we compared the results of the intervention against multiple comparators, which allowed us to consider the results of the intervention within the broader context of the food supply at the time of the intervention and post-intervention. We used an annually updated nutrition dataset that utilises a consistent and standardised method for obtaining nutrition information from products available from Australia supermarkets. This provided us with a reliable dataset across a comprehensive range of products, and the large sample size enhanced statistical power to assess differences in the nutrient quality of products over time and within and across groups. Our analysis, which disaggregated products as matched and unmatched, allowed us to examine nutrient changes from both reformulation and from discontinuation of products and introduction of new product lines.

A limitation of the analyses is that the nutrition information of products was only collected for products that were available in store at four supermarket store locations and, therefore, it is unlikely we have complete coverage of all relevant products. Nevertheless, as the data were collected systematically at four large supermarkets, it is likely the dataset is broadly representative of products available for sale at both time points. Secondly, this was not a controlled trial and, therefore, there are other factors such as company characteristics (size, ownership and market share) that may have contributed to the results seen. However, we have compared the intervention supermarket against three comparators which collectively represent a substantial proportion of the Australian food supply from the same economic and political environment as the intervention supermarket, thereby reducing potential bias that may have been introduced by comparing against just one group. As our paper analysed an Australian-based intervention and compared against other packaged foods sold in Australian supermarkets, there is uncertainty about the broader generalisability of our findings to other countries. Due to a small sample size across some food categories, we did not assess the effect of the intervention at the food category level due to risk of potential statistical conclusion errors. Additional research is required to analyse the impact of supermarket-based interventions at the food category level, particularly as some food categories may be easier to reformulate, which may help to identify effective interventions in the future.

Lastly, as the purpose of this study was to assess the nutrient outcomes arising from the intervention, future qualitative research in the form of a process evaluation is required to understand the extent to which the intervention supermarket used the information provided in the product category reports and how helpful these were in informing changes to their product portfolios. Additional research is also required to understand the barriers faced by supermarkets when undertaking reformulation and formulation of new products and the factors that are considered in the process—for example, the impact on product sales.

## 5. Conclusions

In conclusion, our findings demonstrate that an intervention based on supplying comparative nutrition data to a supermarket retailer was ineffective in achieving consistent improvements to the nutrient content of their private-label products. Relative to comparators, the intervention supermarket achieved a significant reduction in the sodium content of its products over time. However, no improvements were observed for sugar, saturated fat, energy or Health Star Rating. The limited impact of this intervention suggests that there are other factors influencing the decision-making process behind product reformulation, product discontinuation and product innovation such as cost, taste and the impact of product sales. However, additional research is required to explore these factors further. 

## Figures and Tables

**Table 1:** Number (per cent) of all products and specific product types available in each year.

Product Categories	All groups		
	*n* (%)		Change over Time (%)
	2015	2018	
All products	6703 (100)	8428 (100)	+25.7
Bread and bakery products	1344 (20.1)	1663 (19.7)	+23.7
Cereal and grain products	699 (10.4)	837 (9.9)	+19.7
Convenience foods	624 (9.3)	1058 (12.6)	+69.6
Dairy	1069 (15.9)	1383 (16.4)	+29.4
Edible oils and oil emulsions	79 (1.2)	100 (1.2)	+26.6
Fish and fish products	289 (4.3)	309 (3.7)	+6.9
Fruit and vegetables	521 (7.8)	545 (6.5)	+4.6
Meat and meat products	577 (8.6)	853 (10.1)	+47.8
Non-alcoholic beverages	256 (3.8)	235 (2.8)	−8.2
Sauces, dressings, spreads and dips	944 (14.1)	1111 (13.2)	+17.7
Snack foods	266 (4.0)	298 (3.5)	+12.0
Special foods	35 (0.5)	36 (0.4)	+2.9

## Supplementary Materials

The following are available online at https://www.mdpi.com/2072-6643/12/6/1692/s1, Table S1: List of food categories selected as part of the intervention, Table S2: Percentage change in sodium content (mg/100 g) over time by product category, Table S3: Percentage change in sugar content (g/100 g) over time by product category, Table S4: Percentage change in saturated fat content (g/100 g) over time by product category, Table S5: Percentage change in energy content (kJ/100 g) over time by product category, and Table S6: Percentage change in Health Star Rating (stars) over time by product category.
