# Supplementary material for: The Effects of a Supermarket-Based Intervention on the Nutritional Quality of Private-Label Foods: A Prospective Study
Source: Nutrients. 2020 Jun 5;12(6):1692. doi: 10.3390/nu12061692 (PMC7353040; doi:10.3390/nu12061692)
Supplement: Supplementary file 1 [file nutrients-12-01692-s001.pdf]

Table S1. List of food categories selected as part of the intervention

Major Category	Minor Category	Subcategory
1. Bread and bakery products	Biscuits	Plain biscuits, savoury biscuits, sweet filled and unfilled biscuits
	Bread	White bread, wholemeal bread, mixed grain bread, flat bread, pita bread, tortillas, fruit bread, pizza bases, garlic bread, English muffins, crumpets
	Cakes, muffins & pastries	Vanilla/plain cakes, chocolate cake, fruit cakes, lamingtons, Bavarian, Christmas cakes and puddings, cheesecakes, fruit cake mix, vanilla/plain cake mix, chocolate cake mix, pancake mix, sweet pastries, quiche
2. Cereal and grain products	Breakfast cereals	Flavoured oats, flakes with additions, corn flakes, puffed cereals, breakfast biscuits, sweet cereals, muesli with fruit, granola/cluster cereals
	Noodles	Flavoured noodles
	Rice	Ambient flavoured rice and dry packet flavoured rice
	Pasta	Vegetarian and meat/seafood packet pasta
	Cereal and Nut Bars	Cereal and nut bars, cereal-based bars, nut-based bars, puffed bars, baked and filled bars
3. Convenience foods	Pizza	All
	Ready meals	Ambient noodle-based meals, ambient Asian-style meals, pasta-based meals, chilled lasagne, chilled risotto, chilled meat and veg meals, chilled Asian style meals, chilled Asian meals without rice, frozen lasagne, frozen meat and veg meals, frozen Asian style meals, frozen Asian dumpling
	Soup	Canned soup, chilled soup, dry soup mixes
	Sandwiches & salads	Sandwiches, potato salad, coleslaw, pasta salad, couscous-based salad
4. Dairy	Cheese	Parmesan, feta, block cheese, sliced cheese, grated cheese, processed cheese, flavoured cream cheese, ricotta, cream cheese, cottage cheese, other flavoured soft cheese
	Milk	Chocolate flavoured dairy milk, coffee flavoured dairy milk, strawberry flavoured dairy milk, vanilla flavoured dairy milk and other flavoured dairy milk
	Ice-cream	Frozen yoghurt, vanilla ice cream, chocolate ice cream, other sweet ice cream, fruit flavoured ice cream, ice cream cones, choc coated ice cream sticks

	Yoghurt & yoghurt drinks	Drinking yoghurt, flavoured yoghurt, yoghurt with muesli or other non- fruit additions, natural yoghurt, soy yoghurt, dry yoghurt mix, lactose free yoghurt, yoghurt with fruit
5. Edible oils and oil emulsions	Edible oils	Salted butter, margarine
6. Fish and fish products	Processed fish	Canned salmon, canned tuna, coated/battered frozen fish
7. Fruit and vegetables	Fruit	Fruit in juice and syrup
	Nuts & seeds	Salted nuts
	Vegetables	Baked beans, canned asparagus, canned beetroot, canned corn, canned legumes, canned mushrooms, canned tomato, frozen potato chips, frozen hash browns, other frozen potato products
8. Meat and meat products	Processed meat	Bacon, canned chicken, coated/breaded/frozen/chilled meat, meat pies, sausage rolls, meat burgers, salami and cured meat, hot dogs, beef sausages, chicken sausages, pork sausages, sliced chicken, sliced ham, sliced luncheon meat, raw flavoured beef, raw flavoured lamb, raw flavoured chicken, raw flavoured turkey, raw flavoured veal, raw flavoured pork), whole hams
	Meat alternatives	Meat free burgers and flavoured tofu
9. Non-alcoholic beverages	Soft drinks	Sugar sweetened cola, sugar sweetened lemonade, sugar sweetened lemon squash, sugar sweetened creaming soda and other sugar sweetened soft drinks
	Juices	Fruit poppers, nectars, lemon-based juice, sparkling juice
10. Sauces, dressings, spreads and dips	Sauces	Soy sauce, liquid stock, gravy stock, marinades, sweet and sour sauce, Dijon mustard, American style mustard, ambient and fresh tomato-based pasta sauce, fresh pasta sauce with meat, BBQ sauce, chilli and sweet chili sauce, tomato sauce, tomato paste.
	Spreads and dips	Dips, peanut butter, other nut-based spreads, relishes, pickles, chutney
	Mayonnaise & salad dressings	Mayonnaise and salad dressings
11. Snack foods	Crisps & snacks	Corn chips, extruded snacks, salt and vinegar snacks, popcorn and potato crisps
12. Special foods	Breakfast beverages	All

Table S2. Change in sodium content (mg/100g) over time by product category

Product Categories	Year	Intervention			Comparator A			Comparator B			Comparator C		
		n	Sodium content (mg/100g) Mean±SD	Change over time (2018-2015) mg/100g (%)	n	Sodium content (mg/100g) Mean±SD	Change over time (2018-2015) mg/100g (%)	n	Sodium content (mg/100g) Mean±SD	Change over time (2018-2015) mg/100g (%)	n	Sodium content (mg/100g) Mean±SD	Change over time (2018-2015) mg/100g (%)
All products	2015	420	364±372	-39	596	344±271	+48	661	406±426	+21	5026	456±706	+6
	2018	565	325±268	(-10.7%)	899	392±350	(+14.0%)	922	427±417	(+5.2%)	6042	462±701	(+1.3%)
Bread and bakery products	2015	84	315±210	-4	147	314±158	+21	171	343±239	+26	942	437±278	-18
	2018	146	311±155	(-1.3%)	216	335±157	(+6.7%)	198	369±219	(+7.6%)	1103	419±260	(-4.1%)
Cereal and grain products	2015	27	240±355	-114	44	176±176	+17	56	210±184	+36	572	294±542	+40
	2018	27	126±189	(-47.5%)	56	193±168	(+9.7%)	91	246±215	(+17.1%)	663	334±561	(+13.6%)
Convenience foods	2015	86	290±128	+5	40	329±116	-10	24	306±160	+15	474	520±1151	-67
	2018	134	295±115	(+1.7%)	104	319±163	(-3.0%)	71	321±144	(+4.9%)	749	453±859	(-12.9%)
Dairy	2015	31	381±520	+32	96	353±350	+56	119	297±422	+21	823	300±396	-15
	2018	54	413±511	(+8.4%)	106	409±467	(+15.9%)	160	318±408	(+7.1%)	1063	285±377	(-5.0%)
Edible oils and oil emulsions	2015	4	746±397	-133	3	520±151	-82	11	450±118	+37	61	433±169	+1
	2018	3	613±70	(-17.8%)	11	438±148	(-15.8%)	8	487±154	(+8.2%)	78	434±184	(+0.2%)
Fish and fish products	2015	14	340±88	-40	46	359±102	+25	44	404±142	+26	185	399±161	-21
	2018	15	300±57	(-11.8%)	47	384±92	(+7.0%)	47	430±163	(+6.4%)	200	378±138	(-5.3%)
Fruit and vegetables	2015	44	127±176	+49	65	163±130	+2	50	156±165	+86	362	169±176	+36
	2018	55	176±170	(+38.6%)	83	165±151	(+1.2%)	67	242±282	(+55.1%)	340	205±251	(+21.3%)
Meat and meat products	2015	65	649±550	-195	76	561±287	+64	77	800±431	-37	359	855±490	-54
	2018	70	454±228	(-30.0%)	158	625±335	(+11.4%)	126	763±428	(-4.6%)	499	801±462	(-6.3%)
Non-alcoholic beverages	2015	10	5.1±2	+5.7	18	8±2	0	12	7±3	-1	216	9±10	0
	2018	5	10.8±5	(+111.8%)	19	8±3	(0.0%)	11	6±3	(-14.3%)	200	9±11	(0.0%)
Sauces, dressings, spreads and dips	2015	28	514±512	-16	44	506±397	+78	70	698±762	-84	802	780±1184	+66
	2018	25	498±558	(-3.1%)	73	584±679	(+15.4%)	103	614±739	(-12.0%)	910	846±1295	(+8.5%)
Snack foods	2015	27	495±228	-85	17	600±162	-34	23	613±208	+37	199	596±333	-38
	2018	31	410±261	(-17.2%)	26	566±269	(-5.7%)	38	650±280	(+6.0%)	203	558±286	(-6.4%)
Special foods	2015	0	-	-	0	-	-	4	78±5	-3	31	68±22	-10
	2018	0	-		0	-		2	75±7	(-3.8%)	34	58±25	(-14.7%)

Table S3. Change in sugar content (g/100g) over time by product category

Product Categories	Year	Intervention			Comparator A			Comparator B			Comparator C		
		n	Sugar content (g/100g) Mean±SD	Change over time (2018-2015) g/100g (%)	n	Sugar content (g/100g) Mean±SD	Change over time (2018-2015) g/100g (%)	n	Sugar content (g/100g) Mean±SD	Change over time (2018-2015) g/100g (%)	n	Sugar content (g/100g) Mean±SD	Change over time (2018-2015) g/100g (%)
All products	2015	420	9.2±12.4	+0.6	596	10.6±12.7	-1.7	661	12.5±14.1	-1.7	5026	10.2±12.3	-0.7
	2018	565	9.8±13.0	(+6.5)	899	8.9±11.4	(-16.0%)	922	10.8±13.2	(-13.6%)	6042	9.5±11.7	(-6.9%)
Bread and bakery products	2015	84	24.5±16.3	-0.7	147	21.2±16.3	-2.8	171	22.7±17.7	-1.2	942	16.3±16.5	-0.2
	2018	146	23.8±16.2	(-2.9%)	216	18.4±14.9	(-13.2%)	198	21.5±17.8	(-5.3%)	1103	16.1±16.2	(-1.2%)
Cereal and grain products	2015	27	15.1±10.4	+0.9	44	17.4±11.2	-3.2	56	23.3±10.6	-5.7	572	18.3±11.1	-3.2
	2018	27	16.0±8.4	(+6.0%)	56	14.2±12.3	(-18.4%)	91	17.6±11.3	(-24.5%)	663	15.1±11.0	(-17.5%)
Convenience foods	2015	86	3.5±2.7	-0.8	40	4.2±2.8	-1.0	24	3.4±1.9	-0.2	474	3.3±3.7	-0.3
	2018	134	2.7±1.8	(-22.9%)	104	3.2±2.2	(-23.8%)	71	3.2±2.1	(-5.9%)	749	3.0±2.8	(-9.1%)
Dairy	2015	31	7.7±7.9	+0.2	96	10.5±10.2	-0.4	119	11.8±9.7	-0.3	823	10.6±8.8	-0.2
	2018	54	7.9±9.3	(+2.6%)	106	10.1±10.4	(-3.8%)	160	11.5±10.7	(-2.5%)	1063	10.4±9.0	(-1.9%)
Edible oils and oil emulsions	2015	4	1.0±0.0	0.0	3	1.0±0.0	-0.2	11	1.0±0.1	-0.1	61	0.9±0.5	-0.1
	2018	3	1.0±0.0	(0.0%)	11	0.8±0.4	(-20.0%)	8	0.9±0.4	(-10.0%)	78	0.8±0.4	(-11.1%)
Fish and fish products	2015	14	0.7±1.1	+0.5	46	1.5±2.1	0.0	44	2.1±2.0	-0.1	185	1.7±1.7	+0.1
	2018	15	1.2±2.0	(+71.4%)	47	1.5±2.1	(0.0%)	47	2.0±2.1	(-4.8%)	200	1.8±2.1	(+5.9%)
Fruit and vegetables	2015	44	6.7±5.3	-0.4	65	5.6±5.4	+0.4	50	6.3±5.6	-0.9	362	5.8±5.5	+0.1
	2018	55	6.3±5.2	(-6.0%)	83	6.0±5.3	(+7.1%)	67	5.4±4.9	(-14.3%)	340	5.9±7.1	(+1.7%)
Meat and meat products	2015	65	1.4±1.5	-0.1	76	1.6±1.8	+0.5	77	1.3±1.1	+0.2	359	1.4±1.4	+0.3
	2018	70	1.3±1.0	(-7.1%)	158	2.1±2.2	(+31.3%)	126	1.5±1.3	(+15.4%)	499	1.7±1.6	(+21.4%)
Non-alcoholic beverages	2015	10	8.3±4.3	+0.3	18	9.3±4.3	-1.1	12	8.8±4.1	-0.5	216	9.4±3.5	+0.1
	2018	5	8.6±3.7	(+3.6%)	19	8.2±3.6	(-11.8%)	11	8.3±3.6	(-5.7%)	200	9.5±3.1	(+1.1%)
Sauces, dressings, spreads and dips	2015	28	11.7±12.9	-1.4	44	9.2±8.7	+0.1	70	12.2±13.2	-1.8	802	11.1±14.7	-0.5
	2018	25	10.3±10.5	(-12.0%)	73	9.3±10.2	(+1.1%)	103	10.4±11.5	(-14.8%)	910	10.6±13.6	(-4.5%)
Snack foods	2015	27	1.9±1.5	+0.6	17	7.1±13.7	-3.1	23	2.2±1.4	+2.4	199	5.4±10.1	-0.1
	2018	31	2.5±3.2	(+31.6%)	26	4.0±7.9	(-43.7%)	38	4.6±8.0	(+109.1%)	203	5.3±9.5	(-1.9%)
Special foods	2015	0	-	-	0	-	-	4	7.2±0.2	+0.1	31	7.3±1.1	+0.1
	2018	0	-		0	-		2	7.3±0.3	(+1.4%)	34	7.4±1.4	(+1.4%)

Table S4. Change in saturated fat content (g/100g) over time by product category

Product Categories	Year	Intervention n	Intervention Saturated fat content (g/100g) Mean±SD	Intervention Change over time (2018-2015) g/100g (%)	Comparator A n	Comparator A Saturated fat content (g/100g) Mean±SD	Comparator A Change over time (2018-2015) g/100g (%)	Comparator B n	Comparator B Saturated fat content (g/100g) Mean±SD	Comparator B Change over time (2018-2015) g/100g (%)	Comparator C n	Comparator C Saturated fat content (g/100g) Mean±SD	Comparator C Change over time (2018-2015) g/100g (%)
All products	2015	420	4.8±6.5	+0.1	596	5.5±7.0	-0.1	661	5.9±7.1	-0.4	5026	4.7±6.4	+0.1
	2018	565	4.9±6.0	(+2.1%)	899	5.4±7.1	(-1.8%)	922	5.5±6.3	(-6.8%)	6042	4.8±6.4	(+2.1%)
Bread and bakery products	2015	84	7.3±6.4	-1.8	147	6.6±6.1	-0.7	171	8.2±7.4	-1.2	942	5.9±5.8	-0.5
	2018	146	5.5±4.8	(-24.7%)	216	5.9±6.2	(-10.6%)	198	7.0±5.9	(-14.6%)	1103	5.4±5.6	(-8.5%)
Cereal and grain products	2015	27	2.2±1.8	+0.1	44	2.2±2.9	+0.3	56	3.6±2.9	-0.4	572	3.6±3.5	+0.1
	2018	27	2.3±2.3	(+4.5%)	56	2.5±2.4	(+13.6%)	91	3.2±3.3	(-11.1%)	663	3.7±3.4	(+2.8%)
Convenience foods	2015	86	2.5±1.8	-0.3	40	1.8±1.5	+0.2	24	1.2±1.5	+0.7	474	1.9±1.9	-0.1
	2018	134	2.2±1.6	(-12.0%)	104	2.0±1.4	(+11.1%)	71	1.9±2.0	(+58.3%)	749	1.8±1.8	(-5.3%)
Dairy	2015	31	11.1±8.7	+2.0	96	13.2±7.1	+0.3	119	9.6±7.7	+0.6	823	8.8±7.5	+0.2
	2018	54	13.1±8.0	(+18.0%)	106	13.5±7.7	(+2.3%)	160	10.2±7.7	(+6.2%)	1063	9.0±7.6	(+2.3%)
Edible oils and oil emulsions	2015	4	40.9±19.3	-1.3	3	49.3±7.3	-16.5	11	23.5±16.6	-0.5	61	30.4±16.8	-0.4
	2018	3	39.6±24.1	(-3.2%)	11	32.8±21.6	(-33.5%)	8	23.0±14.4	(-2.1%)	78	30.0±17.3	(-1.3%)
Fish and fish products	2015	14	1.4±0.8	+0.5	46	1.2±1.5	+0.3	44	0.9±0.5	+0.2	185	1.2±0.8	0.0
	2018	15	0.9±0.7	(-35.7%)	47	1.5±1.6	(+25.0%)	47	1.1±0.6	(+22.2%	200	1.2±0.9	(0.0%)
Fruit and vegetables	2015	44	2.8±4.0	+0.5	65	1.4±3.2	+0.1	50	1.4±2.5	0.0	362	1.5±2.7	-0.3
	2018	55	3.3±4.2	(+17.9%)	83	1.5±2.8	(+7.1%)	67	1.4±2.6	(0.0%)	340	1.2±2.5	(-20.0%)
Meat and meat products	2015	65	4.5±3.5	+0.2	76	4.4±3.3	+0.3	77	5.5±4.0	-0.3	359	5.2±3.7	-0.1
	2018	70	4.7±3.7	(+4.4%)	158	4.7±3.7	(+6.8%)	126	5.2±4.1	(-5.5%)	499	5.1±3.7	(-1.9%)
Non-alcoholic beverages	2015	10	0.1±0.1	+0.1	18	0.0±0.0	+0.2	12	0.0±0.0	+0.1	216	0.1±0.3	+0.1
	2018	5	0.2±0.4	(+100.0%)	19	0.2±0.4	(-)	11	0.1±0.1	(-)	200	0.2±0.3	(+100.0%)
Sauces, dressings, spreads and dips	2015	28	2.5±3.7	+1.2	44	2.7±3.6	+0.5	70	1.9±2.8	+0.9	802	2.5±3.5	+0.5
	2018	25	3.7±4.1	(+48.0%)	73	3.2±3.3	(+18.5%)	103	2.8±4.3	(+47.4%)	910	3.0±3.9	(+20.0%)
Snack foods	2015	27	4.5±3.5	+1.0	17	7.1±4.8	+0.9	23	9.9±6.1	-1.7	199	6.9±5.3	-0.6
	2018	31	5.5±5.1	(+22.2%)	26	8.0±7.7	(+12.7%)	38	8.2±7.3	(-17.2%)	203	6.3±5.3	(-8.7%)
Special foods	2015	0	-	-	0	-	-	4	0.2±0.0	0.0	31	0.4±0.3	+0.3
	2018	0	-		0	-		2	0.2±0.0	(0.0%)	34	0.7±0.5	(+75.0%)

Table S5. Change in energy content (kJ/100g) over time by product category

Product Categories	Year	Intervention n	Intervention Energy content (kJ/100g) Mean±SD	Intervention Change over time (2018-2015) kJ/100g (%)	Comparator A n	Comparator A Energy content (kJ/100g) Mean±SD	Comparator A Change over time (2018-2015) kJ/100g (%)	Comparator B n	Comparator B Energy content (kJ/100g) Mean±SD	Comparator B Change over time (2018-2015) kJ/100g (%)	Comparator C n	Comparator C Energy content (kJ/100g) Mean±SD	Comparator C Change over time (2018-2015) kJ/100g (%)
All products	2015	420	1101±720	-28	596	1091±657	-2	661	1186±703	-43	5026	1098±722	-24
	2018	565	1129±696	(+2.5%)	899	1089±664	(-0.2%)	922	1143±673	(-3.6%)	6042	1074±699	(-2.2%)
Bread and bakery products	2015	84	1556±413	-39	147	1542±398	-59	171	1734±412	-67	942	1628±406	-44
	2018	146	1517±376	(-2.5%)	216	1483±415	(-3.8%)	198	1667±401	(-3.9%)	1103	1584±419	(-2.7%)
Cereal and grain products	2015	27	1634±129	-30	44	1543±371	-175	56	1671±335	-196	572	1636±335	-99
	2018	27	1604±348	(-1.8%)	56	1368±567	(-11.3%)	91	1475±521	(-11.7%)	663	1537±506	(-6.1%)
Convenience foods	2015	86	592±200	-16	40	620±227	-92	24	466±313	+39	474	556±344	+5
	2018	134	576±243	(-2.7%)	104	528±269	(-14.8%)	71	505±273	(+8.4%)	749	561±351	(+0.9%)
Dairy	2015	31	1002±554	+131	96	1221±430	-15	119	947±498	+67	823	908±520	-5
	2018	54	1133±483	(+13.1%)	106	1206±429	(-1.2%)	160	1014±519	(+7.1%)	1063	903±514	(-0.6%)
Edible oils and oil emulsions	2015	4	2940±194	-277	3	3017±15	-448	11	2273±498	+112	61	2513±528	+2
	2018	3	2663±644	(-9.4%)	11	2569±510	(-14.8%)	8	2385±341	(+4.9%)	78	2515±464	(+0.1%)
Fish and fish products	2015	14	619±195	0.0	46	678±280	-13	44	654±183	+34	185	686±203	+22
	2018	15	619±208	(0.0%)	47	691±262	(+1.9%)	47	688±196	(+5.2%)	200	708±185	(+3.2%)
Fruit and vegetables	2015	44	1053±1102	+117	65	566±816	+118	50	749±921	-38	362	770±942	-139
	2018	55	1170±1154	(+11.1%)	83	684±886	(+20.8%)	67	711±810	(-5.1%)	340	631±758	(-18.1%)
Meat and meat products	2015	65	810±284	-5	76	794±247	+82	77	908±312	-7	359	900±344	+12
	2018	70	805±223	(-0.6%)	158	876±255	(+10.3%)	126	901±355	(-0.8%)	499	912±328	(+1.3%)
Non-alcoholic beverages	2015	10	160±66	+4	18	164±66	-13	12	156±68	+16	216	172±58	0.0
	2018	5	164±26	(+2.5%)	19	151±58	(-7.9%)	11	172±33	(+10.3%)	200	172±51	(0.0%)
Sauces, dressings, spreads and dips	2015	28	986±951	+288	44	834±791	+334	70	799±702	+139	802	867±780	+92
	2018	25	1274±1142	(+29.2%)	73	1168±940	(+40.0%)	103	938±766	(+17.4%)	910	959±822	(+10.6%)
Snack foods	2015	27	2108±181	-43	17	2138±157	-16	23	2159±118	-61	199	2065±175	+7
	2018	31	2065±231	(-2.0%)	26	2122±198	(-0.7%)	38	2098±288	(-2.8%)	203	2072±164	(+0.3%)
Special foods	2015	0	-	-	0	-	-	4	303±6	+3	31	312±34	+12
	2018	0	-		0	-		2	306±8	(+1.0%)	34	324±50	(+3.8%)

Table S6. Change in Health Star Rating (stars) over time by product category

Product Categories	Year	Intervention n	Intervention Health Star Rating (stars) Mean±SD	Intervention Change over time (2018-2015) stars (%)	Comparator A n	Comparator A Health Star Rating (stars) Mean±SD	Comparator A Change over time (2018-2015) stars (%)	Comparator B n	Comparator B Health Star Rating (stars) Mean±SD	Comparator B Change over time (2018-2015) stars (%)	Comparator C n	Comparator C Health Star Rating (stars) Mean±SD	Comparator C Change over time (2018-2015) stars (%)
All products	2015	420	2.9±1.2	0.0	596	2.8±1.2	0.0	661	2.6±1.3	0.0	5026	2.8±1.3	0.0
	2018	565	2.9±1.2	(0.0%)	899	2.8±1.2	(0.0%)	922	2.6±1.2	(0.0%)	6042	2.8±1.2	(0.0%)
Bread and bakery products	2015	84	1.9±1.1	+0.2	147	2.1±1.2	+0.2	171	1.9±1.3	+0.1	942	2.3±1.2	+0.1
	2018	146	2.1±1.1	(+10.5%)	216	2.3±1.2	(+9.5%)	198	2.0±1.2	(+5.3%)	1103	2.4±1.2	(+4.4%)
Cereal and grain products	2015	27	3.9±0.7	+0.1	44	3.7±0.9	-0.1	56	3.3±0.7	0.0	572	3.5±1.2	-0.1
	2018	27	4.0±0.7	(+2.6%)	56	3.6±0.7	(-2.7%)	91	3.3±0.7	(0.0%)	663	3.4±1.2	(-2.9%)
Convenience foods	2015	86	3.5±0.4	0.0	40	3.4±0.4	+0.1	24	3.5±0.2	0.0	474	3.4±0.6	+0.1
	2018	134	3.5±0.4	(0.0%)	104	3.5±0.4	(+2.9%)	71	3.5±0.5	(0.0%)	749	3.5±0.6	(+2.9%)
Dairy	2015	31	2.7±1.1	-0.1	96	2.5±1.1	-0.2	119	2.4±1.3	0.0	823	2.8±1.3	-0.1
	2018	54	2.6±1.2	(-3.7%)	106	2.3±1.2	(-8.0%)	160	2.4±1.2	(0.0%)	1063	2.7±1.3	(-3.6%)
Edible oils and oil emulsions	2015	4	1.1±1.3	+0.4	3	0.7±0.3	+1.3	11	2.5±1.3	-0.2	61	2.0±1.4	0.0
	2018	3	1.5±1.7	(+36.4%)	11	2.0±1.5	(+185.7%)	8	2.3±1.2	(-8.0%)	78	2.0±1.4	(0.0%)
Fish and fish products	2015	14	3.9±0.3	+0.2	46	3.9±0.4	-0.1	44	3.9±0.3	-0.2	185	3.8±0.5	+0.1
	2018	15	4.1±0.3	(+5.1%)	47	3.8±0.4	(-2.6%)	47	3.7±0.5	(-5.1%)	200	3.9±0.4	(+2.6%)
Fruit and vegetables	2015	44	3.8±0.5	-0.1	65	3.7±0.4	+0.2	50	3.7±0.5	0.0	362	3.8±0.5	0.0
	2018	55	3.7±0.7	(-2.6%)	83	3.9±0.5	(+5.4%)	67	3.7±0.7	(0.0%)	340	3.8±0.5	(0.0%)
Meat and meat products	2015	65	2.8±1.2	+0.2	76	2.9±1.1	-0.1	77	2.4±1.2	-0.1	359	2.3±1.2	+0.1
	2018	70	3.0±1.1	(+7.1%)	158	2.8±1.1	(-3.5%)	126	2.3±1.2	(-4.2%)	499	2.4±1.1	(+4.4%)
Non-alcoholic beverages	2015	10	2.3±1.8	-0.6	18	1.4±1.0	+0.5	12	2.9±1.9	+0.1	216	2.4±1.6	-0.9
	2018	5	1.7±1.6	(-26.1%)	19	1.9±1.3	(+35.7%)	11	3.0±1.9	(+3.5%)	200	1.5±1.0	(-37.5%)
Sauces, dressings, spreads and dips	2015	28	3.2±1.1	-0.1	44	3.1±1.1	-0.3	70	2.9±1.2	-0.1	802	2.9±1.2	-0.3
	2018	25	3.1±1.2	(-3.1%)	73	2.8±1.3	(-9.7%)	103	2.8±1.1	(-3.5%)	910	2.6±1.2	(-10.3%)
Snack foods	2015	27	3.1±0.9	-0.4	17	2.3±0.8	-0.1	23	2.6±1.1	-0.7	199	2.7±1.0	-0.3
	2018	31	2.7±1.3	(-12.9%)	26	2.2±1.1	(-4.4%)	38	1.9±0.9	(-26.9%)	203	2.4±1.0	(-11.1%)
Special foods	2015	0	-	-	0	-	-	4	4.5±0.0	0.0	31	4.6±0.3	-0.3
	2018	0	-		0	-		2	4.5±0.0	(0.0%)	34	4.3±0.5	(-6.5%)
